# Practical Notes on Diagnosis and Treatment

**Published:** 1910-12-03

**Authors:** 


### Practical Notes on Diagnosis and Treatment.

### Anti-pneumococcic Serum.

The disappointing results obtained with anti-pneumococcic serum were due to the fact that the anti-bodies formed disappeared rapidly from the blood of the horse used for immunisation. In less than five weeks the very definite agglutinating power of the horse's serum was lost; and all evidence of anti-bodies had gone. On consideration, the result seems only what one would expect from the knowledge on the pathology of human infection. In pneumonia the anti-bodies do not seem to last, and recurrences are probably more common than in any other infective disease. It is probable that with a better knowledge of how to produce the serum more promising results might be expected.—*Mr. A. G. Foulerton.*

### Empyemata.

A point about empyema which is difficult to explain is its greater frequency on the left side than the right, while pneumonia itself is more common on the right side. Speaking from my own experience, I should think that a pleural effusion after pneumonia is far more commonly purulent or sero-purulent than serous. The history of the illness is the most important point of distinction between serous and purulent effusions. If there be a history of illness with an acute onset the effusion is probably purulent and pneumococcic; if there be a history of gradual onset the effusion is probably serous and tuberculous.—*Dr. Hector Mackenzie.*

### Post-operative Sequelæ.

No one should forget after an operation to inquire whether the urine has been passed, and I would warn you that if urine is retained there are few more critical operations in surgery than the passage of a catheter. I do not know anything more tedious than genito-urinary sepsis.—*Mr. C. B. Lockwood.*

### Gout and Tuberculosis.

It is obvious that both of these being protean and ubiquitous, there may be considerable resemblances between their manifestations in certain parts of the body, *e.g.* certain skin lesions, or subacute arthritis. The estimation of the blood-pressure in such cases will often throw a flood of light upon the question of diagnosis, for the gouty cases will always have a well-sustained blood-pressure, whereas the tuberculous will always tend to be below the normal.—*Dr. Leonard Williams.*

### Brachial Neuritis.

The constitutional treatment of this affection is by far the most important, and repeated investigations should be made until the toxic or other cause in operation is identified. Sometimes this is a matter of conjecture, and quinine, arsenic, salicylates, iodides, diaphoretics, and diuretics may be tried in turn. Local treatment is at the best only palliative, among the most successful being dry heat, *e.g.* hot cotton-wool or radiant heat-baths.—*Dr. T. H. Savill.*

